# Abstracts of the 18th Annual Congress of the SBRA, Salvador, BA, 20-23 August 2014

**DOI:** 10.5935/1518-0557.20140015

**Published:** 2014

## O-01. The Predictive Value of Serum Concentrations of Anti-Müllerian Hormone for Oocyte Quality

**A. Iaconelli Jr^1,2^, D.P.A.F Braga^1,2^, A.S. Setti^1,2^, R.C.S. Figueira^1,2^, E Borges Jr.^1,2^**

*^1^Fertility - Centro de Fertilização Assistida*

*^2^Instituto Sapientiae - Centro de Estudos e Pesquisa em Reprodução Assistida*

**OBJECTIVE:** To identify a possible correlation between serum anti-Müllerian hormone (AMH) concentrations and oocyte quality, embryo developmental competence and pregnancy.

**MATERIALS AND METHODS:** A total of 4488 oocytes obtained from 408 patients undergoing ICSI cycles between January 2011 and August 2013, were evaluated. Serum AMH levels were measured prior to the start of each cycle and the concentrations were recorded. The oocyte morphologies were evaluated before ICSI, and the embryos were evaluated on days two, three and five of development. The correlation between the AMH level and cycle’s characteristics and implantation rate were evaluated using a Person correlation and binary regression analyses were performed to evaluate the influence of the AMH level on the oocyte and embryo quality, blastocyst formation and the pregnancy chance.

**RESULTS:** As expected, an inverse correlation between the serum AMH levels and maternal age was noted (CC:-0.279, *P* <0.001). Significant positive correlations between the serum AMH level and the number of aspirated follicles (CC: 0.626, *P* <0.001), number of retrieved oocytes (CC: 0.600, *P* <0.001), number of mature oocytes (CC: 0.588, *P* <0.001), fertilisation rate (CC: 0.595, *P* = 0.048), number of obtained embryos (CC: 0.495, *P* <0.001), number of high-quality embryos (CC: 0.504, *P* <0.001), number of transferred embryos (CC: 0.221, *P* <0.001) and implantation rate (CC: 0,116, *P* = 0.031) were noted. The pregnancy chance was also influenced by the serum concentration of AMH (OR: 1.30, CI: 1.13-1.48, *P* <0.001). The serum AMH level did not influence the embryo quality on days two (OR: 1.01, CI: 0.96-1.06, *P* = 0.732) or three (OR: 0.98, CI: 0.65-2.17, *P* = 0.716) or the chance of blastocyst formation (OR: 0.93, CI: 0.41-1.36, *P* = 0.732, Table 2). However, the oocyte quality was influenced by the AMH level (OR: 0.70, CI: 0.52-0.92, *P* <0.001).

**CONCLUSIONS:** Our findings indicate that the serum AMH levels are a useful predictor of the oocyte quality and pregnancy. Therefore, not only a decreased follicle reserve, but also a lower oocyte quality may responsible for the lower pregnancy chance related to AMH levels.

## O-02. Lipid Profile as a Non-Invasive Tool to Predict Endometrial Receptivity - A Pilot Study

**D.P.A.F Braga^1,2^, E. Lo Turco^3^, E. Cabral^4^, M. Eberlin^4^, A. Iaconelli Jr.^1,2^, E. Borges Jr.^1,2^**

*^1^ Fertility - Centro de Fertilização Assistida*

*^2^Instituto Sapientiae - Centro de Estudos e Pesquisa em Reprodução Assistida*

*^3^Universidade Federal de São Paulo - UNIFESP*

*^4^ThoMSon Mass Spectrometry Laboratory*

**OBJECTIVE:** To make use of the analytical power of mass spectrometry (MS) to characterize the lipid profile of the receptive endometrial fluid of patients undergoing frozen- thawed embryo transfer cycles.

**MATERIALS AND METHODS:** For this study 22 samples of endometrial fluid were collected from patients undergoing hormonal replacement therapy for frozen-thawed embryo transfers. Samples were split into groups according to the pregnancy result: Positive, the Receptive-Endometrium-Group (RE-Group, n=13) and negative, the Non-Receptive-Endometrium-Group (NRE-Group, n-9). To avoid the bias of the embryo quality to the implantation success, the study included exclusively frozen-thawed cycles with blastocyst embryo transfer in which one or two top quality embryos were transferred. The lipid profiles of samples from the RE-Group were compared with the lipid profiles of samples from the NRE-Group, aiming to identify a possible biomarker of the receptive endometrium. Mass spectra fingerprinting were acquired using a 7.2T LTQ FT Ultra-MS, equipped with a chip-based direct infusion nanoelectrospray ionization source. Data were analysed using the Partial Least Square Discrimination Analysis (PLS-DA) combined with variable influence in the projection (VIP) scores and a ROC Curve was constructed.

**RESULTS:** Following the VIP analysis, the most important lipid species were identified. The model detected a significant increase of 14 lipids in the RE-Group including glycerolipids, glycerophospholipids and polyketides. Samples of RE-Group were correctly identified with an 89.0% average probability and an area under the curve of 98.7% was observed.

**CONCLUSIONS:** The MS with ultra-high resolution enables the identification of specific lipids that are differentially represented depending on the endometrial status. Therefore the MS fingerprinting is a valuable, non-invasive tool to predict the endometrial receptivity, however, the study must be performed in a higher number of samples to validate the results.

## O-03. Evaluation of *AMH* and *AMHR2* Polymorphisms, AMH, FSH and Estradiol Serum Levels, and Antral Follicle Counting in Infertile Women and Their Correlation with Human Reproduction Outcomes

**C. Peluso^1^, V. Cavalcanti^1^, G. Gastaldo^1^, D. Christofolini^1^, C. P. Barbosa^1^, B. Bianco^1^**

*^1^Center of Human Reproduction and Genetics - Department of Human Reproduction - Faculdade de Medicina do ABC, Santo André / SP, Brazil*

**OBJECTIVE:** Standard protocol of ovarian hyperstimulation can result in satisfactory response, as for inadequate response which requires dose adjustment of FSH or ovarian hyperstimulation syndrome (OHSS). On the other hand, polymorphisms in *AMH* and *AMHR2* genes seem to affect hormone biological activities, thus affecting follicle recruitment and development, leading to infertility. Here, we aimed to evaluate the G146T/lle49Ser/rs10407022 of *AMH* and A-482G/rs2002555 of *AMHR2* polymorphisms in infertile women underwent assisted reproduction treatment, correlating with AMH, FSH and estradiol serum levels, antral follicle counting and controlled ovarian hyperstimulation (COH) response and assisted reproduction outcomes.

**MATERIALS AND METHODS:** We performed a crosssectional study comprising 186 infertile women that underwent the first cycle of high complexity assisted reproduction treatment. Taqman assay was used for rs10407022 and rs2002555 genotyping. Serum AMH, FSH and estradiol was measured by Elisa assay.

**RESULTS:** The results disclosed that AMH rs10407022 and AMHR2 rs2002555 polymorphisms were not statistically associated with AMH, estradiol and FSH serum levels. The genotypes distribution of these polymorphisms according to COH and human reproduction outcomes did not show a positive association, except for the number of embryos produced (variant allele T and G of the rs10407022 and rs2002555, respectively). Considering COH, AMH can predict OHSS, the minimum value of AMH detected in these patients was almost four times higher compared to the other patients. Regarding AMH serum level and assisted reproduction outcomes we found positive association with antral follicle couting (AFC), follicles visualized at USG, oocytes retrieved and number of frozen embryo. Comparing AFC and AMH as predictor of human reproduction outcomes, the AFC was less effective than serum AMH. However, AFC was positively correlated with OHSS as well as AMH. Consedering βHCG results, neither AMH, AFC or polymorphisms were positively associated with pregnancy rates.

**CONCLUSIONS:** AMH rs10407022 and AMHR2 rs2002555 polymorphisms were positively associated to the number of embryos produced. AMH can be considered a good biomarker to predict OHSS as well as human reproduction outcomes.

**Acknowledgements:** FAPESP for the Master in Science scholarship (#2011/15045-4) and grants (#2011/086811).

## O-04. Effect of Endometriosis on the Functional Ovarian Reserve and on the Reproductive Outcomes of Women Undergoing Assisted Reproductive Techniques

**M.A. Coelho Neto^1^, W.P. Martins^1^, M.A.P. Barbosa^1^, C.O. Nastri^1^, R.A. Ferriani^1^, P.A.A.S. Navarro^1^**

*^1^Setor de Reprodução Humana, Departamento de Ginecologia e Obstetrícia, Faculdade de Medicina de Ribeirão Preto, Universidade de São Paulo*

**OBJECTIVE:** To evaluate the effect of endometriosis on the functional ovarian reserve and on the reproductive outcomes of women undergoing assisted reproductive techniques (ART).

**MATERIAL AND METHODS:** We examined a retrospective cohort (observational study) of women undergoing ART between Jan-2011 and Dec-2012. Only the first cycle of each woman with fresh embryo transfer in the period was considered in the analysis. Women with endometriosis by video laparoscopy and/or ultrasound were compared with all other women. This comparison was also performed in two subgroups, depending on the antral follicle count (AFC): ≤ 6 and ≥ 7. We evaluated age, AFC, number of oocytes obtained, and the clinical pregnancy, live birth and frozen embryo rates.

**RESULTS:** Between 2011 and 2012, 787 different women underwent ART; 214 (27.2%) women diagnosed with endometriosis were compared with 573 women with other causes of subfertility. Considering all women, there is no significant difference in age (34.4±4.3 vs. 34.5±4.5; endometriosis vs. control), clinical pregnancy (22.4% vs. 27.5%, *P* =0.08), and live birth rates (19.1% vs. 22.5%, *P* = 0.16); however the AFC (10.1±8.6 vs. 14.5±10.6; *P* <0.001), the number of eggs obtained (5.6±5.3 vs. 7.3±5.5, *P* <0.001) and frozen embryo rate (32.0% vs. 42.1%, *P* <0.01) were lower. Considering only women with CFA≤6, the age of women with endometriosis was lower (35.2±4.5, N = 96 vs. 37.3±4.5, N = 124, *P* <0.001); however, the number of oocytes obtained (3.2±4.3 vs. 3.6±3.8), the clinical pregnancy (11.5% vs. 13.7%), the live birth (9 4% vs. 8.9%) and the frozen embryo rates (14.6% vs. 14.5%) were similar between groups. Considering only women with CFA≥7, there was a small reduction in the number of oocytes obtained (7.2±5.3 vs. 8.3±5.5, *P* =0.03), however the values were similar for age (33.8±4.0, N = 145 vs. 33.7±4.4, N = 422), clinical pregnancy (29.7% vs. 31.5%), live birth (25.5 % vs. 26.5%) and frozen embryo rates (43.4% vs. 50.2%).

**CONCLUSIONS:** Women with endometriosis have a poorer functional ovarian reserve. However, they have similar reproductive results when compared with women having similar AFC and other similar causes of subfertility.

## O-05. *In Vitro* Maturation of Human Preantral Ovarian Follicles in a Tridimentional Culture System

J.K. Rodrigues^1,2^, R.M. Marinho^1,2,3^, A. M. M. Cota^1^, A.J.A. Wainstein^3^, J.P.J. Caetano^1,2,3^

*^1^Pró-Criar Medicina Reprodutiva*

*^2^Rede Brasileira de Oncofertilidade/ Brazilian Oncofertility Consortium - reBOC/BOC*

*^3^Instituto de Pós-graduação/ Faculdade de Ciências Médicas de Minas Gerais*

**OBJECTIVE:** To evaluate the in vitro development of human ovarian preantral follicles, and the viability of the clinical application of the technique.

**MATERIAL AND METHODS:** It was performed an in vitro three-dimensional (3D) follicular culture of human ovarian preantral follicles, obtained from a small sample of fresh ovarian tissue fragment harvested from a 36 year old patient with breast cancer prior to chemotherapy, and used for cryopreservation for future fertility. Two secondary follicles (125-225 µm) were encapsulated in alginate and cultured for 28 days in MEM culture medium supplemented with insulin, transferrin, synthetic serum substitute and Follicle Stimulating Hormone. The survival, growth and antrum formation were monitored weekly.

**RESULTS:** Two preantral follicles were isolated, due to the small sample size and lack of follicles in the ovarian tissue that was collected. One of follicles survived for a period of 7 days, and the other was maintained in culture for 28 days. The first follicle did not show growth, and gradually decreased its diameter. The second increased its diameter in 2.3 times, with 217.6 µm in diameter on the first day and 499.1 µm at day 28 of culture. None of them showed antrum formation.

**CONCLUSIONS:** We report for the first time in Brazil, the culture of human preantral follicles in a 3D culture system. The study shows promising results, proving the possibility of establishment of an in vitro follicular growth model. The study has great clinical importance for the preservation of ovarian tissue of young cancer patients, who for ethical and/or medical reasons can not go undergo ovarian stimulation and oocyte cryopreservation. The establishment of the technique of ovarian tissue cryopreservation and subsequent in vitro follicle maturation could prevent the implantation of malignant cells in patients treated for cancer and allow to obtain a larger number of oocytes for fertilization.

## O-06. Validation of NGS Technology for the Detection of Embryonic Segmental Aneuploidies

**M. Riboldi^1^, M. Vera^2^, C.E. Michel^3^, N. al-Asmar Piñar^4^, C. Simon^2,5,6^, C. Rubio^2^**

*^1^IGENOMICS Brasil Laboratório de Medicina Genética Ltda, São Paulo, Brasil*

*^2^IGENOMICS Espanha S.L, Parque Científico, Valencia, Espanha*

*^3^Illumina, Cambridge, UK*

*^4^IGENOMICS USA, Miami, Estados Unidos*

*^5^Fundación Instituto Valenciano de Infertilidad (FIVI)/INCLIVA, Valencia, Espanha*

*^6^Department of Obstetrics and Gynecology, School of Medicine, Stanford University, CA, Estados Unidos*

**OBJECTIVE:** To evaluate the capability of next-generation sequencing (NGS) to detect segmental aneuploidies in trophectoderm biopsies and to define the concordance rate with the results obtained with the current platform of array comparative genomic hybridization (aCGH).

**MATERIALS AND METHODS:** Amplified DNA from trophectoderm biopsies in which segmental aneuploidies were identified by aCGH in a CCS cycle were selected. A total of 88 segmental aneuploidies were detected on amplified DNA using aCGH. Subsequently, surplus amplified DNA samples were reanalyzed by NGS. For aCGH, DNA was labeled, co-hybridized in 24sure arrays and analyzed by BlueFuse Multi software. For NGS, a library was generated from double stranded DNA (dsDNA) and loaded into a MiSeq instrument.

**RESULTS:** Segmental aneuploidies were defined as partial gains or losses of partial chromosome segments with a minimum size of 10 Mb. Thirty-four (34) segmental aneuploidies were detected in carriers of reciprocal translocations, these embryos were defined as the control group with known potential unbalances. The remaining 54 segmental events were identified in blastocysts from couples with a normal karyotype and were considered the study group. In total, the concordance rate between NGS and aCGH was 98.9%.

In the control group, the concordance rate was 100%, whereas in the study group it was 98.1%. Only one discrepancy was observed between NGS and aCGH or an embryos in which NGS showed a normal result and aCGH showed a partial gain for the long arm of chromosome 17 (15.2Mb).

**CONCLUSIONS:** NGS allows the detection of segmental aneuploidies in human blastocyst with the same efficiency as aCGH. Studies like this are essential for the development of appropriate software that allows the efficient translation of NGS into the CCS programs.

## O-07. Ultrastructural Evaluation of Eutopic Endometrium of Infertile Patients with and without Minimal/ Mild Endometriosis During the Window of Implantation: A Pilot Study

**M.G. Da Broi^1^, C.V. Rocha Jr^1^, F. M. Faria^2^, A.R. da Silva^3^, R. Ferriani^1^, P. Navarro^1^**

*^1^Setor de Reprodução Humana, Departamento de Ginecologia e Obstetrícia, Faculdade de Medicina de Ribeirão Preto (FMRP) - USP*

*^2^Serviço de Patologia Clínica (SERPAT), Hospital das Clínicas de Ribeirão Preto, HCFMRP-USP ^3^Departamento de Patologia, FMRP-USP*

**OBJECTIVE:** The etiopathogenesis of endometriosis-related infertility still needs elucidation. One of the mechanisms involved may be an alteration in endometrial receptivity. Pinopodes are apical structures of endometrial epithelial cells which are related to embryo implantation. An asynchrony in its expression may be associated with the infertility in these patients. The aim of this study was to evaluate the cell pattern, including the presence and development stage of pinopodes (P), in eutopic endometrium of infertile patients with and without minimal/mild endometriosis (ME) during the window of implantation (WOI), using scanning electron microscopy (SEM).

**MATERIALS AND METHODS:** endometrial biopsies from 10 infertile patients (5 with ME and 5 controls (C) with male and/or tubal factor of infertility, without E according to diagnostic laparoscopy evaluation) were taken between days 20 and 24 of the cycle with histological dating performed by 2 independent readers according to the Noyes’ criteria. Samples were processed and analyzed by SEM. Cell types (microvilli cells - MC, ciliated cells - CC and P) observed in each analyzed image were identified and quantified, and the development stage of P was classified. The average of the fields analyzed for each sample and the frequencies of cell types in the two groups were calculated.

**RESULTS:** The E group showed a P coverage area of 61.48% (530/862), a CC area of 6.27% (54/862) and a MC coverage of 32.25% (278/862). Among P cells, 8.49% (45/530) were developing, 13.77% (73/530) were fully developed and 77.74% (412/530) were regressing. In the C group, 65.1% (526/808) of the areas were covered by P, 5.82% (47/808) by CC and 29.08% (235/808) by MC. Among the P cells, 16.92% (89/526) were classified as developing, 34.22% (180/526) as fully developed and 48.86% (257/526) as regressing.

**CONCLUSIONS:** The predominance of regressing P and the lower percentage of developed and developing P in E group during the WOI should suggest an asynchrony in the expression of these structures in the endometrium of infertile women with ME which might be involved in impaired endometrial receptivity and in minor endometriosis-related infertility. These data need further confirmation in larger sample size studies well powered.

**Support:** FAPESP (2011/17614-6), Brazil.

## O-08. Is the Number of Oocytes Retrieved Associated with IVF Outcome?

**N.I. Zavattiero-Tierno, V.M. Lopes, J.P.B. Brasileiro, E.F. Duarte, M. Roller, L.G.L. Nolêto**

*Instituto Verhum, Brasilia, DF*

*Hospital Universitario de Brasilia, UnB, Brasilia, DF*

**OBJECTIVE:** To evaluate the association of rate of ongoing pregnancy and number of MII oocytes retrieved.

**MATERIAL AND METHODS:** This is a retrospective cohort study (01-12/2013) of patients aged ≤35 years undergoing in vitro fertilization. Patients were divided into 2 groups: Group A: 5-9 oocytes MII retrieved and Group B: ≥10. Variables included: age, infertility factor, BMI, endometrial thickness and duration and type of hypophysary blockage (HB). We considered ongoing pregnancy the proportion of patients with at least one fetus showing heart beat on 12 week ultrasound over the total number of transfers performed.

The statistical package Statatm (Statacorp, USA) was used to conduct all analyses and calculate relative risks (RR) of absence of ongoing pregnancy and 95% confidence interval (95% CI).

**RESULTS:** A total of 73 patients fulfilled all eligibility criteria; with 48 in group A and 25 in group B. The mean age in Group A was 32.3 years (95% CI: 31.4-33.1), BMI 23.4 (95% CI: 21.8-24.9), endometrial thickness 11.1 mm (95% CI: 10.5-11.8), duration of stimulus 11.3 days (95% CI: 10.7-11.9), duration of HB with an agonist of 12.3 days (95% CI: 11.5-13.1) and of an antagonist of 10.4 days (95% CI: 9.6-11.3) and mean number of MII oocytes retrieved 6.8 (95% CI: 6.4-7.2). In Group B the corresponding mean age was 31.8 (95% CI: 30.7-32.8), BMI 23.3 (95% CI: 20.4-26.3), endometrial thickness 10.0 mm (95% CI: 9.2-10.9), duration of stimulus 11.5 days (95% CI: 11.0-12.1), duration of HB with an agonist of 11.8 days (95% CI: 11.0-12.6), and of an antagonist of 11.1 days (95% CI: 10.1-12.1) and mean number of MII oocytes retrieved 12.7 (95% CI: 11.3-14.1). The ongoing pregnancy rate was 41.7% in Group A and 36.0% in Group B (*P*=0.8).

The relative risk for absence of ongoing pregnancy did not differ by Group (RR =0.9; 95% CI: 0.5-1.6), even after stratification for all other variables.

**CONCLUSIONS:** The rate of ongoing pregnancy by group was not associated with the number of MII oocytes retrieved (<10 and ≥10 oocytes) with a trend toward better result in Group A. We plan to reevaluate with a larger sample.

## O-09. Efficacy of a Human Stem Cell Medium on the Development of Mice Embryos

**C. Lauritzen^1,2^, R. A. Lima^1^, M. Gomes^1^, M. Frajblat^1^**

*^1^Instituto de Biofísica Carlos Chagas Filho, Universidade Federal do Rio de Janeiro*

*^2^Fundação de Amparo à Pesquisa do Estado do Rio de Janeiro*

**OBJECTIVE:** In the field of assisted reproductive medicine there is a constant demand for new human embryo culture media to increase embryo quality and increase the possibility of single embryo transfer. The aim of this study was to assess the efficacy of a human embryonic stem cell media using an animal model of embryo development.

**MATERIAL AND METHODS:** Two-cells mouse embryos were cultured in three commercially available embryos culture medium (Single Step Medium™ IRVINE; Continuous Single Culture™ Irvine; Human Tubal Fluid Global^®^, LIFE TECHONOLOGY), supplemented with 10% fetal bovine serum. The fourth media was a embryoninc stem cell one produced by the Laboratório Nacional de Células-Tronco Embrionárias (LANCE-) named Brastem^®^. Embryos were cultured for four days in microdrops of 50ul coverd with mineral oil. Embryo development was recorded until blastocyst stage.

**RESULTS:** The 4 cell (80,8%) and blastocyst (77,5%) rates were not different among the groups. However, blastocyst hatching rate was decreased in the Brastem group (66,7; 56,7; 70,0 e 46,7% respectively).

**CONCLUSIONS:** These results indicate that Brastem media has the potential to also be used as embryo development media in mice and needs further testing to confirm this potential.

## O-10. Evaluation of Survival Rate and Clinical Pregnancy of Vitrified Blastocysts D5 Versus D6 in Cycles with Single Embryo Transfer

**I.H. Yoshida^1^, P.C. Rodrigues^1^, F.E. Mizrahi^1^, E. Tognotti^1^, L.O. Tso^1^, N.E. Busso^1^**

*^1^Projeto ALFA - Aliança de Laboratórios de Fertilização Assistida*

**OBJECTIVE:** To evaluate if the day on which blastocysts are freezing (day 5 or day 6) interferes with the survival and clinical pregnancy rate in cycles of single embryo transfer.

**MATERIAL AND METHODS:** A retrospective cohort study. Infertile women who underwent assisted reproduction treatment without getting pregnant in the fresh cycle were included. The surplus blastocysts were vitrified on day 5 or day 6 after IVF/ICSI for another attempt, using the Irvine Scientific^®^ medium and Cryotop^®^ blades. The devitrification occurred between January 2012 and April 2014 with single embryo transfer. They were divided into two groups: group 1: blastocysts were vitrified on D5 (n = 57) and group 2: blastocysts were vitrified on D6 (n = 58). The groups are homogeneous female, mean age 33.8 years to D5 and 34.8 for D6. The ß-hCG was quantified 15 days after embryo transfer. The presence of clinical pregnancy gestational sac with visible heartbeat on transvaginal ultrasonography performed 15 days after the ß-hCG. These data were correlated between groups using Chi-Square and the statistical package SPSS.

**RESULTS:** The survival of blastocysts in Group 1 (D5) was higher than Group 2 (D6) (86% and 65.5%, respectively) (*P* = 0.011). There was no difference between the clinical pregnancy rate per thawed in Group 1 than in Group 2 (36.8% and 20.7%, respectively) (*P* =0.056), but not statistically significant.

**CONCLUSIONS:** The blastocysts had frozen on D5 shows the survival rate higher than D6, so the day of embryo cryopreservation interferes on survival results without significantly influence on pregnancy rate.

## O-11. Extending the Embryo Culture Until the 6^Th^ Day of Development Increases the Rate of Aneuploidy in Blastocysts?

**M.M. Piccolomini^1^, M. Nicolielo^1^; N.G. Nobrega^1^, E.L.A. Motta^1,2^, P. Serafini^1,3^, J.R. Alegretti^1,2.^**

*^1^Huntington Medicina Reprodutiva - São Paulo*

*^2^Departamento de Ginecologia - Universidade federal de São Paulo (UNIFESP) - São Paulo*

*^3^Centro de Reprodução Humana - Universidade de São Paulo (USP) - São Paulo*

**OBJECTIVE:** Assess the rates of aneuploidy in expanded blastocysts biopsied on day five (D5) and sixth (D6) development in patients undergoing in vitro fertilization (IVF) and analysis by CGH-array.

**MATERIALS AND METHODS:** Between January 2013 and March 2014, 1,433 expanded blastocysts of 667 patients who underwent fertility treatment with IVF + CGHa were analyzed. The blastocysts were biopsied between days 5 and 6 of embryonic development. The women were divided into 4 groups according to age group randomly chosen (A = ≤ 34 years, B = 35-37 years, C = 38-40 years and D = ≤ 41 years). Statistical analysis included the chi-square test was used.

**RESULTS:** In D5, 1035 blastocysts were biopsied and 277 (26.76%) euploid and 758 (73.24%) aneuploid; while in D6, 398 blastocysts were biopsied resulting in 101 (25.38%) euploid and 297 (74.62%) aneuploid no statistically significant difference (*P*=0.594). The age of patients in both groups was similar (*P*> 0.50). Evaluation of aneuploidy related to the different maternal age groups revealed no significant differences. In group A 208 blastocysts were analyzed in D5, 129 aneuploid (62%) and 64 in D6, 37 aneuploid (58%) (*P*=0.546). Group B, with 207 blastocysts evaluated in D5, 150 aneuploid (72%) and 69 in D6, 52 aneuploid (75%) (*P*=0.638). In group C, 348 blastocysts analyzed in D5, and 253 aneuploid (73%) and 113 in D6, 87 aneuploid (77%) (*P*=0.368). Finally, group D, 272 blastocysts analyzed D5 being aneuploid 226 (83%) and 152 in D6, 121 aneuploid (80%) (*P*=0.372).

**CONCLUSIONS:** The data presented in this study showed that the extended embryo culture to the D6 does not increase the rates of aneuploidy in blastocysts. The occurrence of a blastocyst euploid be is not necessarily associated with more accelerated kinetics of embryonic growth and even the very ability to deploy a receptive endometrium. Similarly, the cultivation extended to D6 appears to maintain the biological processes of embryonic growth and maintenance of embryonic ploidy.

## O-12. Role of Endometrial Receptivity Array (ERA) in the Orientation of Personalized Embryo Transfer (pET)

**M. Riboldi^1^, M. R. Alonso^2^, E. Gomez-Sanchez^2^, A. Rincón-Bertolín^2^, B. Coprerski^1^, C. Simon^2,3^**

*^1^IGENOMICS Brasil Laboratório de Medicina Genética Ltda, São Paulo, Brasil*

*^2^IGENOMICS Espanha S.L, Parque Científico, Valencia, Espanha*

*^3^Fundación Instituto Valenciano de Infertilidad (FIVI)/ INCLIVA, Valencia, Espanha*

**OBJECTIVE:** Demonstrate clinical value of ERA in patients undergoing assisted reproductive treatments to guide a pET.

**MATERIAL AND METHODS:** 60 patients were selected; including 48 performed a treatment with oocyte donation (OD) while the other 12 underwent in vitro fertilization treatment with their own oocytes (IVF). The patients were divided into 4 groups according to the number of repetitive implantation failures (RIF): Group 0 = 0 failures, Group 1 = 1 failure, Group 2 = 2 failures and Group 3 = ≥3 failures. All patients, for the realization of the ERA test, received the same endometrial preparation, beginning on the 1st day of the cycle: Day 1-10: E2 = 6mg/day and day 11-16: P4 = 800mg/day with realization of endometrial biopsy by Pipelle, on the 5th day of action of progesterone.

**RESULTS:** 60 patients with Non-receptivity (NR) result in the 1st ERA teste were studied, the mean BMI was 22.3±2.2 and mean RIF was 2.2±2.0. Mean age = 40.3±4.7 between OD patients and = 34.6±3.0 IVF patients. The analysis of 238 genes by ERA test observed that 90% (54/60) of patients had endometrium in pre-receptive status (PRE) while 10% (6/60) was observed post-receptive (POS) [G0: PRE=92%/POS=8%; G1: PRE=92%/POS=8%; G2: PRE=100%/POS=0; G3: PRE=85%/POS=15%]. With the completion of 2nd endometrial biopsy to validate previous results from displacement of the deployment window, enabled 92% (55/60) of patients obtain results Receptive (R) [G0: R=92%; G1: R=92%; G2: R=100%; G3: R=88%] while only 8% (5/60) the result maintained NR. These results enabled the realization of a pET with implantation rates in G0: 39%; G1 35%, G2: 40%; G3: 45% a total of 41% (43/105) and clinical pregnancy rates were G0: 88%; G1 29%, G2: 80%; G3: 69% a total of 67% (24/36). Biochemical pregnancy rates were G0: 12%; G1: 57%, G2: 20%; G3: 12% and total of 11% rate of abortion.

**CONCLUSIONS:** The realization of the ERA test allowed us to find the exact day of Endometrial Receptivity corresponding the Window of Implantation and thus, performing a pET increasing rates of Pregnancy and Implantation in all patients regardless of their historic and type of treatment.

## O-13. Ratio of Progesterone (on hCG Day) to Number of Aspirated Oocytes as a Prognostic Tool in IVF Cycles

**M. Roque^1^, M. Valle^1^, F. Guimarães^1^, M. Sampaio^1^, S. Geber^1^**

*^1^Origen - Centro de Medicina Reprodutiva*

**OBJECTIVE:** Progesterone elevation (PE) during controlled ovarian stimulation (COS) is associated with a decreased probability of pregnancy in fresh embryo transfer and this deleterious effect may vary according to ovarian response. The main objective of this study was to establish a ratio of *P* levels on hCG day to the number of aspirated oocytes (P/Oocyte ratio) and to evaluate whether this ratio was associated with in vitro fertilization (IVF) outcomes.

**MATERIALS AND METHODS:** A prospective observational cohort study was conducted between January 2012 and June 2013. A total of 335 patients submitted to COS with gonadotropin-releasing hormone antagonist protocol and cleavage-stage day 3 fresh embryo transfers were included; all had *P* levels ≤1.5 ng/mL on hCG day. The P/Oocyte ratio was calculated as [*P*(ng/mL)/number of aspirated oocytes], *P* levels measured on the day of final oocyte maturation. The statistical analysis was performed using Student’s t test, the chi-square test, and linear regression models. Receiver operating characteristics (ROC) analysis was conducted to establish the most efficient cut-off value for the P/Oocyte ratio to discriminate between successful and unsuccessful IVF outcomes, and the highest value of the area under the curve (AUC) was determined. A *P* value of <0.05 was considered statistically significant. The main outcome measure was ongoing pregnancy rate.

**RESULTS:** Using ROC, we established a cut-off level of 0.09 for the P/Oocyte ratio. The sensitivity (71%), specificity (66%), and AUC (0.761; 95% CI 0.711-0.812) of the test showed that it was a good prognostic test. Ongoing pregnancy rates and implantation rates were found to be significantly higher in patients with P/Oocyte ratio ≤0.09 than those with >0.09 (54% vs 20%, *P*<0,0001 and 34.4% vs 12.6%, *P*<0.0001, respectively.

**CONCLUSIONS:** Even in a selected group of patients without PE (*P* levels ≤1.5 ng/mL), the *P*/Oocyte ratio is a good prognostic test for IVF outcomes that can correlate the *P* levels with the ovarian response. It would be better to use this ratio than a single cut-off value of P levels as a prognostic tool.

## O-14. Vitrified Human Embryos from in Vitro Maturation (IVM) Lead to the Birth of Healthy Children

**N.P. Oliveira^1^, G.N. Frantz^1^, C.G. Dutra^1^, C.G. Basso^1^, M.F. FORTIS^1^, N. Frantz^1^**

*^1^Nilo Frantz - Centro de Reprodução Humana*

**INTRODUCTION:** IVM can be an advantageous technique when applied to PCOS Polycystic Ovarian Syndrome) patients. The oocytes are retrieved from antral follicules of unstimulated ovaries, specially preventing hyperstimulation syndrome. Apart from its role as reproductive treatment, IVM has emerged as a promising tool for emergency fertility preservation, since it spares the use of gonadotrophins and can be performed flexibly in either follicular or luteal phase.

**CASE REPORT:** A 34-year-old old patient with PCOS, amenorrhea, BMI (body mass index) 28.5, and tubal factor. Her husband has low count spermatozoa. She underwent the first IVM in 2009, transferred 3 fresh embryos and got pregnant giving birth to a healthy boy weighing 3.3 kg. In 2013, the patient returned for another IVM treatment. From the 10 oocytes picked up, 6 became metaphasis II after 30 hours in IVM Medicult media and were inseminated by ICSI. All of them fertilized, resulting in 6 embryos graded day 3 as: 1G1 (best grade), 2G2, 1 G3 and 2G4 (worst grade). The embryos were vitrified because she failed to develop an adequate endometrium for transfer. In the next cycle, the endometrium was prepared using estrogen and progesterone and the two best embryos were warmed up and transferred. She became pregnant and after 36 weeks gave birth to a healthy girl weighing 2.7 kg.

**COMMENTS:** IVM may be an alternative technique to be considered when dealing with PCOS patients. Although clinical outcomes are currently inferior compared with conventional hormone driven ART (Artificial Reproductive Techniques), it does apply in some cases while preventing hyperstimulation risks. Thus, embryos obtained by IVM can also be vitrified with successful results.

## O-15. Expression of *Dmc1* and *Rec8* in Mice: Evidence of Oogenesis During Mammal´S Adult Life

**D.B. Dentillo^1^, F.L.S. Zerbini^1^, C.C.P. Ribas^1^, R.M. Reis^1^, A.C.J.S. Rosa e Silva^1^, M.F. de Sá^1^**

*^1^Departamento de Ginecologia e Obstetrícia da FMRP-USP*

**OBJECTIVE:** to investigate whether meiosis-specific genes, expressed in pre-diplotene phases (which is believed to be restricted just to the intrauterine life in female mammals), are expressed in oavaries of adult mice.

**MATERIAL AND METHODS:** we isolated RNA from ovary samples of twenty 65 years old C57Bl/6 mice and cDNA molecules were obtained by reverse transcription. Then we performed the polymerase chain reaction (PCR) - 95^o^C for 10 minutos, followed by 30 cycles of: 95^o^C for 15 seconds; 55^o^C for 15 seconds; and 72^o^C for 20 seconds - with specific primers for Rec8 e Dmc1 genes, both of them expressed in pre-diplotene phases of prophase I of meiosis. As reaction positive control, we used the endogenous gene that codify β-actin (Actb). Testicle samples were used as positive controlo of the experiment, as meiosis take place continuously in males´ gonads; and kidney samples were used as negative controlo f the experimente, once meiosis occurs exclusively in the gonads. The results were visualized in agarose gel after electrophoresis.

**RESULTS:** Rec8 and Dmc1 genes, as well as Actb, were amplified (PCR positive reaction) in testicle and ovarian samples; but in kidney samples just Actb was amplified. Notably, the expression of Rec8 and Dmc1 genes was higher in the testicles, when compared to the ovaries.

**CONCLUSIONS:** the results indicate that initial phases of meiosis, as so oogeneisis, are occuring in ovaries of adult mice (experimental model), what represents a paradigm shift on the reproductive biology of mammals. The evidence of oogenesis during mammals´ adult life can radically change the female infertility treatments and new protocols for fertility preservation can be developed from it.

## O-16. L-Carnitine and N-Acetyl-Cysteine Supplementation To *In Vitro* Maturation Media Reduces Oocyte Meiotic Damage Induced by Incubation in Follicular Fluid from Infertile Patients with Mild Endometriosis

**V.S.I. Giorgia^1^, M.G. Da Broi^1^, B.T.G.M. Jianini^1^, C.C.P. de Paz^2^, R.A. Ferriani^1^, P.A.A.S. Navarro^1^**

*^1^Departamento de Ginecologia e Obstetrícia, Faculdade de Medicina de Ribeirão Preto, Universidade de São Paulo*

*^2^Departamento de Genética, Faculdade de Medicina de Ribeirão Preto, Universidade de São Paulo*

**OBJECTIVE:** To assess the impact of follicular fluid (FF) from infertile women with mild endometriosis(ME) on oocyte spindle and chromatin and to evaluate the potential protective effect of L-carnitine(LC) and/or N-acetyl-cysteine(NAC) against deleterious substances present in the FF utilized during in vitro maturation(IVM) in a bovine model.

**MATERIAL AND METHODS:** We performed an experimental study. FF samples were obtained from 22 infertile women undergoing ovarian stimulation for intracytoplasmic sperm injection [11 with ME (EFF) and 11 with tubal and/or male factor of infertility(CFF)], pooled, and utilized in 9 IVM experiments with immature bovine oocytes (IBO). IBO were submitted to IVM divided in 9 groups: without FF (NF), EFF, CFF, EFF + NAC (ENAC), CFF + NAC (CNAC), EFF + LC (ELC), CFF + LC (ECLC), EFF + NAC + LC (E2Ao), CFF + NAC + LC (C2Ao). After 22-24 h of IVM, oocytes were denuded, fixed, stained for morphological visualization of both microtubules and chromatin, and analyzed by confocal microscopy. Data were analyzed by Poisson distribution.

**RESULTS:** A total of 1934 oocytes were fixed and 1572 were analyzed. The percentage of normal MII oocytes was significantly lower in the EFF (51,35%), ENAC (62,22%) and E2Ao (61,40%) groups compared to NF and all CFF groups. The percentage of normal MII oocytes was similar comparing NF (86,36%) with all control groups [CFF (83,52%), CNAC (80,95%), CLC (90,22%), C2Ao (81,03%)] and ELC (80,61%). In the groups with addition of control FF, only LC increased the percentage of normal MII oocytes. And in the groups with addition of mild endometriosis FF, the addition of NAC, LC, and both of them increased the percentage of normal MII oocytes, but LC was significantly superior to NAC and the 2Ao.

**CONCLUSIONS:** FF from infertile women with ME may promote meiotic damage of in vitro matured bovine oocytes, which is minimized or completely prevented by co-administration of antioxidants, especially LC. Our data suggest oxidative stress is involved in the worsening of oocyte quality in ME. We question whether the use of LC as a supplement in patients with ME may be a novel approach to improve fecundity.

**Financial funding:** FAPESP (process number: 2012/15070-1), Brasil.

